# Combination of Pilose Antler Extract and Hydroxytyrosol Enhances Bone Mineral Density in Both Animals and Postmenopausal Women

**DOI:** 10.1002/fsn3.70402

**Published:** 2025-06-12

**Authors:** Xichi Ma, Yun Ma, Xilin Ma, Zubing Zhang, Yuan Li

**Affiliations:** ^1^ Bayi Orthopaedic Hospital Chengdu China; ^2^ Yiping Medical Science & Technology Development Co. Ltd Chengdu China; ^3^ Hehui Pharmaceutical Technology Co., Ltd Chengdu China

**Keywords:** bone health, hydroxytyrosol, olive extract, osteoporosis, pilose antler, postmenopausal

## Abstract

Osteoporosis, characterized by reduced bone mineral density (BMD) and impaired bone microarchitecture, imposes a significant global health and economic burden, particularly on postmenopausal women. This study investigated the efficacy of a novel supplement that combines pilose antler extract and hydroxytyrosol (Ruiling capsule) in improving bone health in animal and human trials. In an osteoporotic rat model, Ruiling supplementation significantly increased BMD and bone calcium content, particularly at medium doses, surpassing untreated controls and restoring values to levels comparable with non‐osteoporotic rats. In a parallel randomized, double‐blind, placebo‐controlled clinical trial, postmenopausal women who received Ruiling capsules for 32 weeks showed marked improvements in lumbar‐spine and femoral‐neck BMD, bone turnover markers, antioxidant enzyme activities, and reduced low back pain compared to the placebo group. No adverse effects were observed, suggesting that modulation of oxidative stress may underpin the bone‐protective effects. These findings highlight the potential of combining pilose antler extract and hydroxytyrosol to address the multifactorial osteoporosis pathogenesis. The Ruiling capsule represents a safe, non‐pharmacological intervention that offers both structural and functional benefits and could contribute to the long‐term management of osteoporosis in aging populations.

## Graphical abstract

Osteoporosis poses a significant health and economic burden globally, particularly in postmenopausal women. This study investigated the efficacy of a novel supplement combining pilose antler extract and hydroxytyrosol in improving bone health through animal and human trials. The supplement was found to be a safe, non‐pharmacological intervention that offers both structural and functional benefits, with implications for long‐term management of osteoporosis in aging populations.

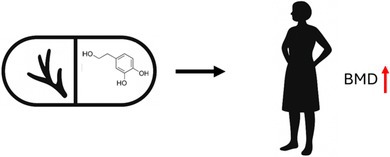

## 1 Introduction

Osteoporosis is characterized by decreased bone mineral density (BMD) and deterioration of bone microarchitecture, resulting in an increased risk of fragility fractures as the main clinical consequence of the disease. (Kanis et al. 2019, 1994; Lorentzon and Cummings 2015). Epidemiological studies have reported that the global prevalence of osteoporosis among men and women is 11.7% and 23.1%, respectively (Salari et al. 2021). Osteoporotic fractures impose a high disease burden and lead to significant health care costs (Rashki Kemmak et al. 2020). In Canada, Europe, and the USA alone, the direct annual cost of treating osteoporotic fractures of people is estimated to be between 5000 and 6500 billion USD (Rashki Kemmak et al. 2020). Because the risk of osteoporosis and osteoporotic fracture increases with age (Kanis 2002), both health and economic burdens caused by osteoporosis fractures are expected to escalate as the global population is aging (Rowland 2009). In China, the annual number of osteoporotic fractures is projected to increase by 135% from 6.9 million in 2020 to 16.2 million by 2024 due to the aging population, with drastically increased economic burden associated with these fractures (Cui et al. 2021).

Postmenopausal women are particularly susceptible to osteoporosis (Cosman et al. 2014; Lei et al. 2023), primarily because the abrupt fall in systemic estrogen removes multiple restraints on bone resorption (Manolagas et al. 2013). Estrogen deficiency up‐regulates RANKL, down‐regulates its decoy receptor osteoprotegerin (OPG), prolongs osteoclast survival, and unleashes pro‐resorptive cytokines such as TNF‐α, IL‐1, IL‐6, and IFN‐γ from osteoblast‐lineage and immune cells (Eastell et al. 2016). It also converts quiescent memory T‐cells into IL‐17 and TNF‐α secreting effector cells (Cenci et al. 2000; Tyagi et al. 2012), further amplifying RANKL expression and osteoclastogenesis. Concomitantly, decreased estrogen weakens antioxidant defenses (superoxide dismutase, glutathione peroxidase), creating chronic oxidative stress in which reactive oxygen species (ROS) stimulate osteoclast differentiation (Manolagas 2010), trigger osteocyte and osteoblast apoptosis (Cruzoé‐Souza et al. 2009) and suppress Wnt/β‐catenin signaling in osteoblast progenitors (Seibel et al. 2013). These immune‐inflammatory and redox‐driven insults, together with age‐related cellular senescence and diminished SIRT1 activity in bone‐forming cells, tilt mesenchymal‐stem‐cell commitment away from osteogenesis toward adipogenesis (Sasaki et al. 2020). The net result is imbalanced bone remodeling, and rapid net bone loss occurs after menopause, primarily in cancellous bone in the first 5 years after menopause and subsequently in both cortical and trabecular bone space at a slower pace (Manolagas et al. 2013; Zhivodernikov et al. 2023).

Because menopause is inevitable, reducing post‐menopausal bone loss and maintaining BMD are critical for women's long‐term well‐being. In addition to lifestyle‐based preventive approaches, including adequate calcium, vitamin D intake, increased levels of physical activity and exercise, smoking cessation, and reduced alcohol consumption (Cosman et al. 2014), current pharmacotherapy for osteoporosis falls into two main categories: anti‐resorptives (reduce bone turnover) and anabolics (bone forming) (Langdahl 2021). Bisphosphonates, including alendronate, risedronate, ibandronate, and zoledronate, are first‐line anti‐resorptives (Russell 2011). Denosumab, a RANKL antibody, is used as a bisphosphonates alternative and has been shown to reduce bone turnover and increase BMD (Cummings et al. 2009). Raloxifene, a selective estrogen receptor modulator, is approved for the prevention and treatment of postmenopausal osteoporosis (Chen et al. 2019). Bone‐forming therapies include parathyroid hormone and parathyroid hormone‐related peptide, which act via the PTH1 receptor (Hattersley et al. 2016), recombinant human PTH 1–34 (teriparatide) (Neer et al. 2001), and the humanized sclerostin antibody romosozumab (Padhi et al. 2011).

Epidemiological studies have shown that the Mediterranean diet, which features high olive oil consumption, is associated with a lower incidence of chronic, age‐related conditions, including osteoporosis (Keys 1995; Keys et al. 1986). Hydroxytyrosol is a phenolic compound extracted from the olive tree (Puel et al. 2008; Wichers et al. 2000) with great antioxidant capacity (Martínez et al. 2018), and is considered safe as a novel food ingredient by the European Food Safety Authority (EFSA) (EFSA Panel on Dietetic Products, Nutrition and Allergies, et al. 2017). Previous research has reported that consumption of hydroxyltyrosol led to the prevention of inflammation‐induced bone loss in ovariectomised (OVX) rats (Puel et al. 2008, 2004). Additionally, hydroxyltyrosol has been demonstrated to confer a bone protecting effect through promoting osteoblast differentiation and inhibition of osteoclast activity (Hagiwara et al. 2011). Taken together, the established bone health promoting activity and the safety of hydroxyltyrosol make it a potentially attractive candidate for dietary interventions aimed at maintaining or improving bone health, particularly in vulnerable populations such as postmenopausal women.

In parallel, traditional medicine systems have long recognized certain animal‐derived products for their bone‐supportive properties (Apaza et al. 2003; Hao et al. 2023; Liu et al. 2024). Pilose antler/velvet antler, a component of traditional Chinese medicine, is reputed to improve the immune system, physical strength, and sexual function (Gilbey and Perezgonzalez 2012; Kawtikwar et al. 2010; Sui et al. 2014; Xia et al. 2022). It contains bioactive components including growth factors (Lai et al. 2007), collagen (Jeon et al. 2012), polypeptides (Chen et al. 2007) and glycosaminoglycans (Takeda‐Okuda et al. 2019) that may enhance osteoblast proliferation, matrix mineralization, and overall skeletal integrity (Linkhart et al. 1996; Shi et al. 1996). Recent studies have indicated that supplementation with pilose antler extracts improves bone density and mechanical strength in animal models by increasing the expression of bone morphogenetic protein‐2 (BMP‐2) and collagen I (COL‐1) (Ren et al. 2019) and by reducing the expression of extracellular signal‐regulated kinase 1 (ERK‐1), c‐Jun N‐terminal kinase (JNK), and matrix metalloproteinase‐9 (MMK‐9) (Liu et al. 2023).

Although the individual benefits of hydroxytyrosol and pilose antler extract on bone health have been established, their combinational effect has not been investigated. Given the multifactorial etiology of osteoporosis, including encompassing hormonal imbalances, oxidative stress, chronic inflammation, and impaired bone remodeling, a combined approach targeting multiple pathological drivers could provide more robust beneficial outcomes (Manolagas and Jilka 1995). The rationale for combining hydroxytyrosol and pilose antler extract lies in their complementary modes of action: hydroxytyrosol's potent antioxidative and anti‐inflammatory effects alongside pilose antler's osteogenic and bone remodeling support. This dual‐action strategy may yield cumulative improvements in BMD, bone microarchitecture, and overall skeletal integrity.

This study therefore investigated the efficacy of combined supplementation with pilose antler extract and hydroxytyrosol (termed as Ruiling capsule) in improving BMD and related bone parameters in both animal models and postmenopausal women. By exploring their integrated effects, we aimed to identify new non‐pharmacological interventions for osteoporosis prevention and management. Ultimately, these findings could support the development of functional dietary formulations that help maintain bone health, enhance quality of life, and reduce the long‐term healthcare burden associated with osteoporosis.

## 2 Material and Methods

### 2.1 Animal Study

#### 2.1.1 Experimental Animals and Housing Conditions

Sixty female Sprague–Dawley (SD) rats at the Specific Pathogen‐Free (SPF) grade were supplied by Chengdu Dashuo Biotechnology Co. Ltd. (License No.: SCXK(Chuan)2013‐24). The animals were housed in barrier‐level individually ventilated cage (IVC) systems approved by the Sichuan Province Experimental Animal Management Committee (License No.: SYXK(Chuan)2013‐011). Standard chow was supplied by Chengdu Dashuo Biotechnology Co. Ltd. Throughout the experiment, the rats had free access to food and water. The housing temperature was maintained at 20°C–23°C with a relative humidity of 55%–66%.

#### 2.1.2 Experimental Methods in Animal Study

After acclimatization on a basal diet, the rats were randomly assigned to five groups based on body weight: a sham‐operated group, a model control group, and three groups receiving different doses of Ruiling (*n* = 12 per group). For the sham‐operated group, anesthesia was induced by intraperitoneal injection of 3% pentobarbital sodium. Under strict aseptic conditions, bilateral dorsal incisions were made near the lumbar vertebrae to access the abdominal cavity, and a small portion of the small intestinal mesentery was removed. Hemostasis was carefully achieved before closing the incisions in layers.

For the model control group (control) and the three Ruiling dosage groups, the same aesthetic and aseptic procedures were followed. Bilateral dorsal incisions were similarly made to enter the abdominal cavity medially, and both ovaries were completely removed. The incisions were closed in layers after hemostasis had been ensured.

On the third day post‐surgery, the sham‐operated and model control groups were administered distilled water by oral gavage. The three Ruiling dosage groups received solutions corresponding to 140 mg/kg body weight (bw) (low dose), 280 mg/kg bw (medium dose), and 840 mg/kg bw (high dose). To prepare the solutions, 1.4 g, 2.8 g, and 8.4 g of Ruiling capsule material were each dissolved in distilled water and brought to a final volume of 100 mL, yielding the required concentrations for 140 mg/kg bw, 280 mg/kg bw, and 840 mg/kg bw, respectively. All animals received a dose volume of 10 mL/kg bw by oral gavage once daily for 90 consecutive days. Body weights were recorded weekly, and the gavage volume was adjusted accordingly.

At the end of the experimental period, the animals were euthanized. The right femur was excised, dried in an oven at 105°C until constant weight was achieved, and then weighed to determine bone mass. The left femur bone mineral density (BMD) was assessed using a small animal X‐ray system (Bruker In Vivo DXS Pro). The calcium content in the right femur was quantified using atomic absorption spectrophotometry.

#### 2.1.3 Statistical Analysis in Animal Study

Statistical calculations were done by using the medical statistical software PEMS3.1 edition developed by the Statistics Teaching Section of Huaxi Hospital. Differences between the model group and the sham‐operated group were assessed using the t‐test. For comparisons of various indicators between each dosage group and the model group, analysis of variance (ANOVA) was employed. If the data did not meet the assumptions for ANOVA, a nonparametric rank‐sum test was used.

### 2.2 Human Study

#### 2.2.1 Study Design

This randomized, double‐blind, placebo‐controlled, parallel clinical trial was conducted at Bayi Orthopedic Hospital. A total of 120 postmenopausal women with osteoporosis were equally allocated into two groups: an observation (intervention) group and a control group, each receiving either Ruiling capsules plus calcium carbonate (intervention) or placebo plus calcium carbonate (control). The entire study duration was 32 weeks (8 months), with assessments conducted at baseline and at the end of the treatment period.

#### 2.2.2 Study Participants

120 eligible postmenopausal women with osteoporosis were recruited and randomly assigned (1:1 ratio) to the observation group (*n* = 60) or the control group (*n* = 60).

Inclusion criteria were: (1) *T*‐score ≤ −2.5 SD at the axial skeleton or distal one‐third of the radius based on dual‐energy x‐ray absorptiometry (DXA) results; (2) postmenopausal for ≥ 2 years; (3) age 46–65 years; (4) baseline low back pain with a Visual Analog Scale (VAS) score ≥ 4; and (5) provision of written informed consent.

Exclusion criteria included secondary osteoporosis, recent use of osteoporosis medications, for example, bisphosphonates therapy, calcium or vitamin D supplement, malignancies, severe systemic diseases, psychiatric disorders, known allergies to the investigational products, and abnormal liver or kidney function.

Participants who withdrew consent, were lost to follow‐up, demonstrated poor adherence (intake outside 80%–120% of prescribed doses), or experienced severe adverse events necessitating unblinding were considered withdrawn. Misdiagnosed cases, those meeting exclusion criteria after enrolment, and participants without any treatment or data were excluded from efficacy analyses but retained for safety assessments if applicable.

#### 2.2.3 Procedures

Following randomization and baseline assessments, the observation group received Ruiling capsules (2 capsules per dose) plus calcium carbonate (1 capsule per dose), twice daily. The control group received identical placebo capsules (2 capsules per dose) plus calcium carbonate (1 capsule per dose), twice daily. Ruiling capsules (Batch No.: 230110) and the matching placebo (Batch No.: 230115) were manufactured by Chengdu Yiping Pharmaceutical Technology Development Co. Ltd. Calcium carbonate (Batch No.: 221004) was obtained from Liaoning Kangchen Pharmaceutical Co. Ltd.

All participants continued their assigned treatment regimen for 8 months (32 weeks). No other osteoporosis treatments were permitted, but medications required for comorbid conditions could be continued and recorded. Baseline and 32‐week follow‐up visits included assessments of pain (VAS), bone mineral density (BMD), and bone metabolism markers. Safety evaluations, including general physical exams, blood and urine tests, ECG, and imaging, were performed at baseline and at 32 weeks.

To maintain blinding, the placebo matched the active product in appearance, odor, and packaging. A third party not involved in the study assigned random numbers and prepared coded study boxes. Emergency unblinding was permitted only under critical clinical conditions, and any such cases were handled as withdrawals in the efficacy analysis.

#### 2.2.4 Biochemistry

Biochemical assessments were conducted at baseline and after 32 weeks of treatment. Bone metabolism markers included β‐crossLaps (β‐CTx), procollagen type I N‐terminal propeptide (P1NP), and 25‐hydroxyvitamin D (25(OH)D), measured via electrochemiluminescence assays. Oxidative level markers including malondialdehyde (MDA), superoxide dismutase (SOD) and glutathione peroxidase (GSH‐Px) were measured to assess the efficacy of Ruiling capsule in anti‐oxidation.

Safety‐related biochemical parameters included complete blood count, urinalysis, fasting blood glucose, liver enzymes, renal function tests, as well as sex hormone levels. Breast and uterine ultrasound examinations were also performed.

#### 2.2.5 Outcomes

##### 2.2.5.1 Primary Efficacy Outcomes (Bone Health)

- Low Back Pain (VAS): A 0–10 cm VAS scale was used, with higher scores indicating more severe pain. Measurements were taken at baseline and after 32 weeks of treatment.
- Bone Mineral Density (BMD): BMD at the lumbar spine (L2–L4) and right femoral neck was measured using DXA at baseline and 32 weeks.
- Bone Metabolism Markers: Levels of β‐CTx, P1NP, and 25(OH)D were assessed at baseline and 32 weeks.

##### 2.2.5.2 Secondary Efficacy Outcomes (Anti‐Oxidant Activity)

The antioxidation activity of Ruiling capsule is assessed by measurement of the serum level of MDA, SOD, and GSH‐Px.

##### 2.2.5.3 Safety Outcomes

General physical examinations, liver and kidney function tests, blood and urine analyses, and imaging studies (e.g., breast and uterine ultrasound) were evaluated at baseline and at 32 weeks to assess tolerability and safety.

#### 2.2.6 Treatment Compliance and Adverse Events

Compliance was evaluated by counting returned capsules at each follow‐up visit. Good compliance was defined as consuming 80%–120% of the prescribed dose. Investigators maintained detailed records of the number of study capsules dispensed, consumed, and returned, as well as any adverse events (AEs). All AEs were recorded on case report forms, and participants with severe or intolerable AEs were withdrawn from the trial.

#### 2.2.7 Statistical Analysis

After data validation and database lock, all analyses were performed using SPSS 25.0 (IBM Corp., Armonk, NY). Continuous variables were expressed as mean ± standard deviation (SD). Between‐group differences were evaluated by ANOVA, followed by post hoc q‐tests if ANOVA showed significance. Paired *t*‐tests assessed within‐group changes from baseline to 32 weeks. For non‐normally distributed data or heterogeneous variances, rank‐sum or t‐tests were employed. Ordinal data were analyzed using Ridit analysis. A *p*‐value < 0.05 was considered statistically significant.

## 3 Results

### 3.1 Animal Trial

#### 3.1.1 Effect of Ruiling Capsule on Body Weight and Bone Measurement in OVX Rat

**FIGURE 1:**
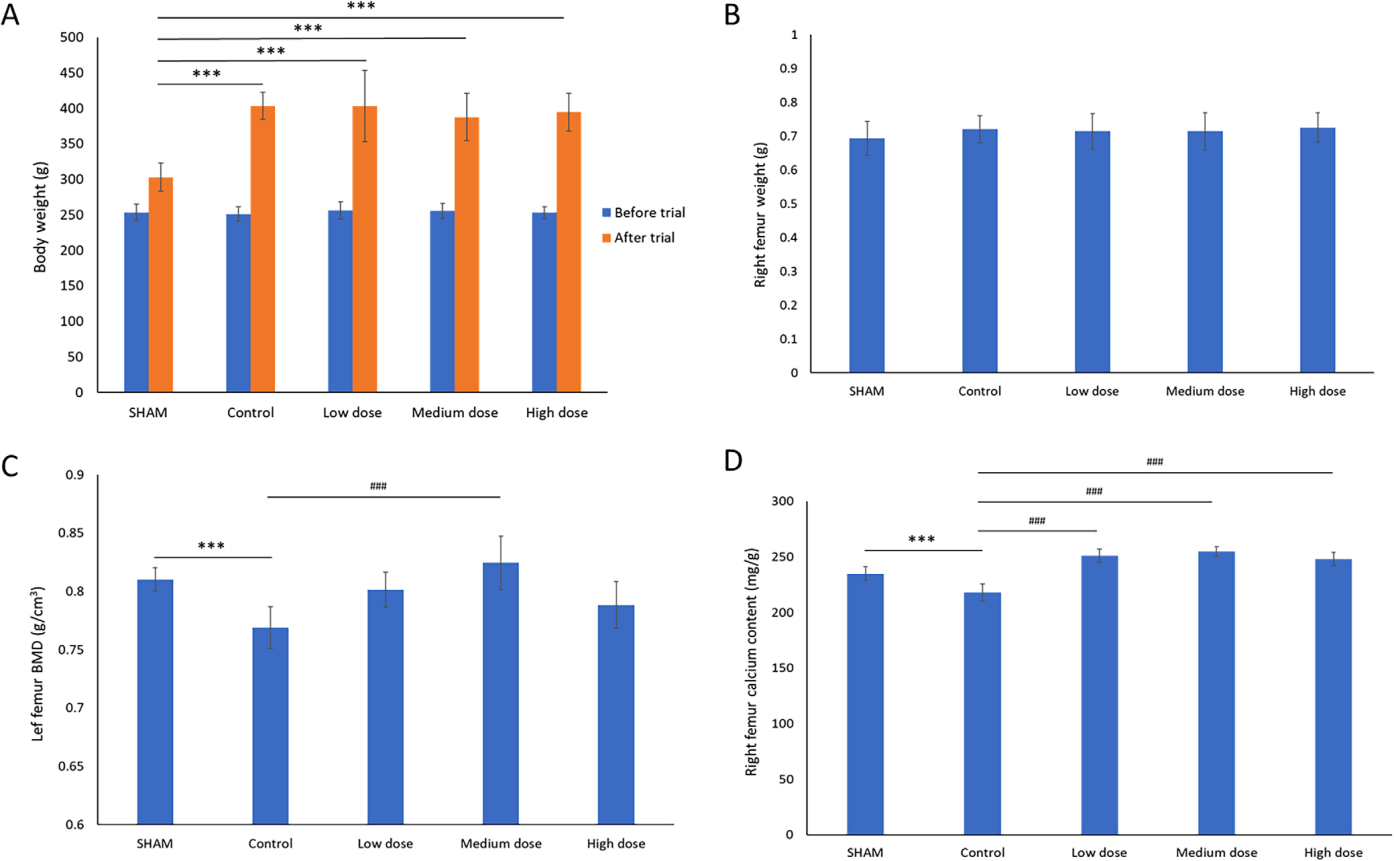
Effects of combined supplementation with Ruiling capsule on body weight, right femur weight, left femur bone mineral density (BMD), and right femur calcium content in rats (*n* = 12 per group). (A) Changes in body weight before and after the 90‐day trial period in sham‐operated (SHAM), untreated osteoporotic (Control), and low‐, medium‐, or high‐dose supplementation groups. The blue bars represent initial body weight and the orange bars represent body weight at the end of the trial. (B) Right femur weight at the end of the experiment. (C) Left femur BMD measured by dual‐energy X‐ray absorptiometry after the treatment period. (D) Right femur calcium content determined at the end of the trial. Low, medium, and high doses correspond to 140 mg/kg bw dosage, 280 mg/kg bw dosage, and 940 mg/kg bw dosage groups. Data are presented as mean ± SD. ***p < 0.01 versus SHAM; ###*p* < 0.01 versus Control. One‐way ANOVA followed by Tukey's multiple‐comparison test for panels B and D; two‐way repeated‐measures ANOVA with Šídák correction for panel A.

The effect of Ruiling capsule on rat body weight showed in Figure 1A, all groups exhibited increased body weight over the 90‐day trial. The SHAM group increased from 253.5 ± 11.4 g at baseline to 302.8 ± 19.9 g at the end of the trial. In contrast, the Control group showed an increase from 251.3 ± 9.9 g to 403.3 ± 18.9 g, significantly higher than SHAM (*p* < 0.01), consistent with previous publication that OVX female rats eat more and gain more weight than intact rats (Blaustein and Wade 1976; Geary and Asarian 1999; Tarttelin and Gorski 1971). The groups receiving low, medium, and high doses of Ruiling capsule supplementation displayed final body weights of 403.4 ± 50.2 g, 387.4 ± 33.4 g, and 394.6 ± 26.7 g, respectively. While these values suggest weight gains similar to the Control group, no statistical significance was indicated among the treatment groups relative to Control in terms of body weight changes.

Right femur weights at the end of the trial were comparable across all groups (Figure 1B). The SHAM group had an average femur weight of 0.693 ± 0.05 g, while the Control group measured 0.72 ± 0.04 g. The low‐, medium‐, and high‐dose groups had femur weights of 0.714 ± 0.052 g, 0.714 ± 0.055 g, and 0.725 ± 0.044 g, respectively. Although treatment groups tended to show slightly higher femur weights compared to SHAM, no significant differences were observed.

Left femur BMD measurements revealed distinct differences among the groups (Figure 1C). The SHAM group maintained the highest BMD value at 0.8101 ± 0.01 g/cm^3^. The Control group, in contrast, exhibited a reduced BMD of 0.7688 ± 0.018 g/cm^3^, indicating a significant decline (*p* < 0.01) relative to SHAM. Notably, medium‐dose supplementation yielded a BMD of 0.8244 ± 0.023 g/cm^3^, demonstrating a significant (*p* < 0.01) improvement compared to Control and surpassing even the SHAM baseline. Although the low‐dose (0.8015 ± 0.015 g/cm^3^) and high‐dose (0.7883 ± 0.02 g/cm^3^) groups showed improved BMD relative to Control, only the medium‐dose achieved a level of significance, as indicated in Figure 1C.

Right femur calcium content was measured at the end of the trial (Figure 1D). The SHAM group had a calcium content of 234.86 ± 6.32 mg/g, while the Control group's value dropped significantly to 217.91 ± 7.82 mg/g (*p* < 0.01). All supplemented groups showed marked improvements in femoral calcium content: low dose 250.82 ± 6.03 mg/g, medium dose 254.98 ± 4.25 mg/g, and high dose 248.06 ± 6.13 mg/g. All dosage groups demonstrated significant (*p* < 0.01) enhancement compared to Control, indicating that administration of Ruiling capsule effectively restored bone mineralization levels in OVX rats.

### 3.2 Human Trial

#### 3.2.1 Baseline Characteristics

**FIGURE 2:**
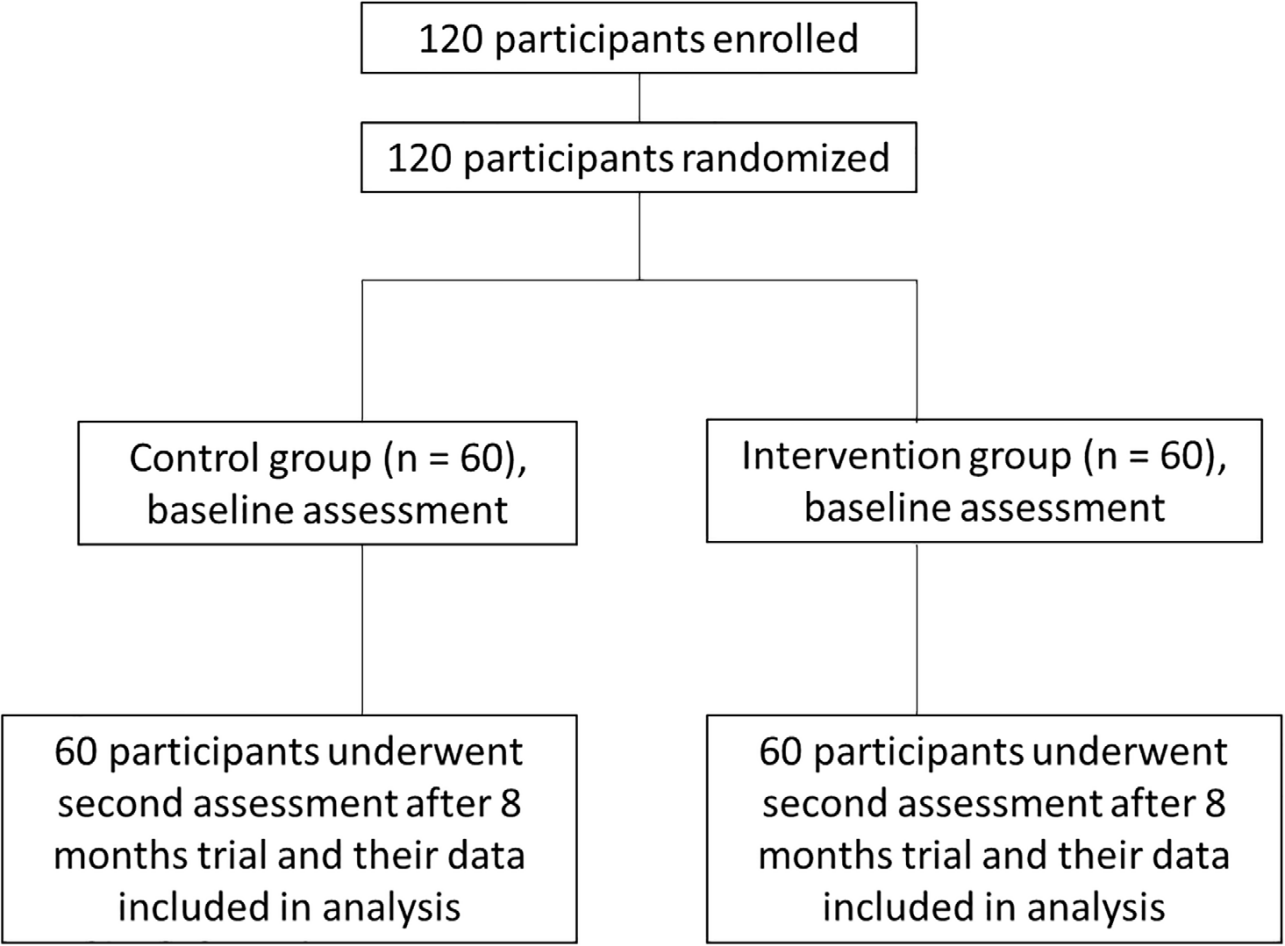
Study participant flow chart. 120 participants were enrolled for the study and randomly divided into two groups (60 each). Participants in each group (*n* = 60) were going through baseline assessment. Participants in the control group were given a placebo and a calcium carbonate capsule; participants in the intervention group were administered Ruiling capsule and a calcium carbonate capsule. Participants who finished the trial underwent a series of physical and bone health tests after 8‐month trial.

A total of 120 postmenopausal osteoporotic patients were enrolled and randomized into two groups (Treatment group: *n* = 60; Control group: *n* = 60) as shown in the study flowchart in Figure 2. No significant differences were observed between the two groups with respect to baseline demographic variables (age, years since menopause, height, body weight, and BMI), vital signs (heart rate, blood pressure), or baseline efficacy measures (low back pain score, lumbar spine and femoral neck BMD, P1NP, β‐CLx, and 25‐hydroxyvitamin D levels) (*p* > 0.05). These findings indicate that the two groups were comparable at the start of the study (Table 1).

**TABLE 1:** Baseline characteristics of the participants.

	Control group (*n* = 60)	Intervention group (*n* = 60)
Age	56.73 ± 4.26	56.97 ± 3.89
Years since menopause	6.30 ± 3.66	7.03 ± 3.45
Height (cm)	154.3 ± 4.14	155.87 ± 5.16
Weight (kg)	57.79 ± 4.12	56.66 ± 3.78
BMI (kg/m^2^)	24.30 ± 2.21	23.39 ± 2.25
Back pain score (VAS)	5.57 ± 1.41	5.63 ± 1.24
BMD: L2–L4 (g/cm^2^)	0.65 ± 0.09	0.65 ± 0.09
BMD: femoral neck (g/cm^2^)	0.62 ± 0.08	0.61 ± 0.07
P1NP (μg/L)	49.83 ± 5.97	50.26 ± 6.03
β‐CTx (ng/L)	540.16 ± 23.46	539.45 ± 24.83
25 (OH)D (ng/mL)	26.41 ± 7.83	25.74 ± 7.39

*Note:* Data are presented as mean ± SD.

#### 3.2.2 Low Back Pain Scores

**FIGURE 3:**
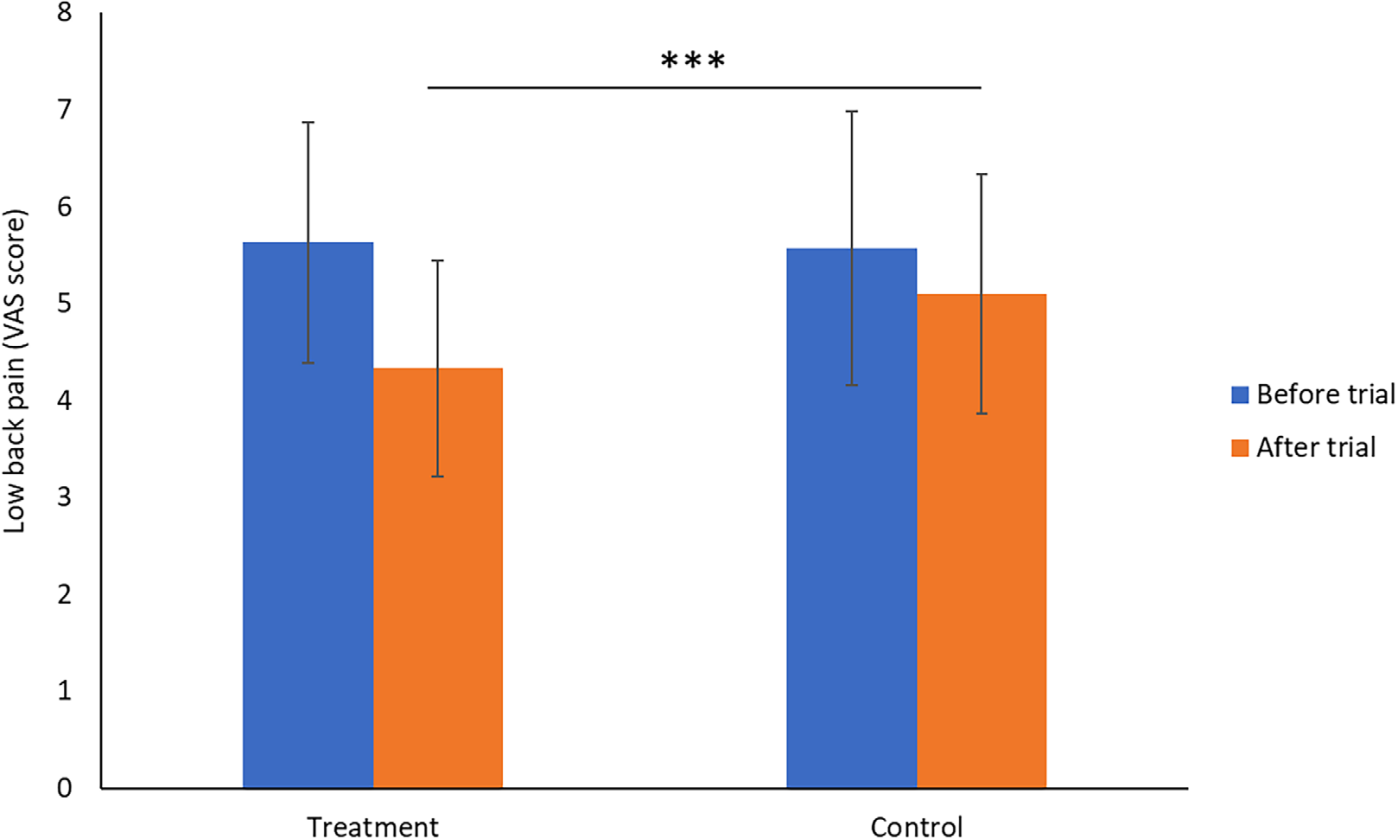
Efficacy of Ruiling capsule on low back pain as assessed by the Visual Analogue Scale (VAS) before and after the trial. Participants were divided into a control group (*n* = 60) and a treatment group (*n* = 60) receiving Ruiling capsules. Data are presented as mean ± SD. Statistical comparison after the trial indicates that the treatment group experienced significantly lower VAS scores compared to the control group (***p < 0.01).

The efficacy of Ruiling capsule on participants' low back pain was assessed and result showed in Figure 3. Prior to treatment, there was no significant difference in low back pain (VAS) scores between the two groups (*p* > 0.05). After 32 weeks of treatment, both groups showed a reduction in pain scores, but the decrease was more pronounced in the treatment group after trial compare to the control group after the trial (*p* < 0.01). Specifically, the treatment group's VAS score declined from 5.63 ± 1.24 to 4.33 ± 1.11, whereas the control group's score changed from 5.57 ± 1.41 to 5.10 ± 1.23.

#### 3.2.3 Bone Mineral Density (BMD)

**FIGURE 4:**
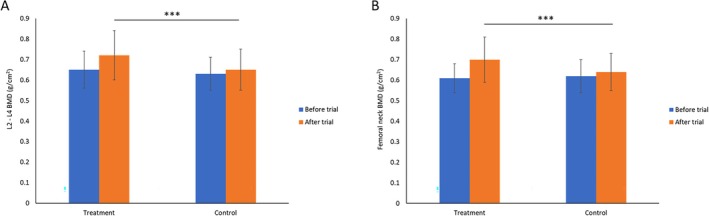
Efficacy of Ruiling capsule on bone mineral density (BMD) in the lumbar spine (L2–L4; A) and the femoral neck (B) of participants before and after the trial. Participants were allocated into a control group (*n* = 60) and a treatment group (*n* = 60) receiving Ruiling capsules. Data are presented as mean ± SD. Statistical comparison following the trial shows that the treatment group exhibited significantly higher BMD than the control group (***p < 0.01).

The efficacy of Ruiling capsule on improving participants' BMD in L2–L4 (Figure 4A) and femoral neck (Figure 4B) was assessed and result showed in Figure 4. Baseline BMD at L2–L4 and the right femoral neck did not differ significantly between the two groups (*p* > 0.05). After 32 weeks of treatment, the treatment group exhibited significantly higher BMD values at both sites compared to the control group (*p* < 0.01). For example, the mean femoral neck BMD in the intervention group improved from 0.61 ± 0.07 g/cm^2^ to 0.70 ± 0.11 g/cm^2^, whereas the control group's BMD increased only from 0.62 ± 0.08 g/cm^2^ to 0.64 ± 0.09 g/cm^2^. A similar pattern was observed in the L2–L4 region (Figure 4A). The corresponding *T*‐score is calculated accordingly (Xue et al. 2021) and shown in Table 2. Participants in both groups are considered to have osteoporosis as their *T*‐scores are < −2.5. After the 32‐week intervention, participants in the intervention group who received Ruiling capsule plus calcium carbonate demonstrated an improvement in *T*‐scores at both the L2–L4 (−2.91 to −2.33) and femoral neck (−2.9 to −2.0) sites compared to their baseline values. In contrast, the control group, which received placebo plus calcium carbonate, showed less improvement. These results suggest that the treatment administered to the treatment group effectively enhanced bone density over the study period.

**TABLE 2:** T‐score of participants in intervention and control group before and after trial.

	Intervention group (*n* = 60)		Control group (*n* = 60)	
	Before trial	After trial	Before trial	After trial
L2–L4	−2.91 ± 0.75	−2.33 ± 1.00	−3.08 ± 0.67	−2.92 ± 0.83
Femoral neck	−2.90 ± 0.70	−2.0 ± 1.10	−2.8 ± 0.80	−2.6 ± 0.90

*Note:* Data are presented as mean ± SD.

#### 3.2.4 Bone Metabolism Markers

**FIGURE 5:**
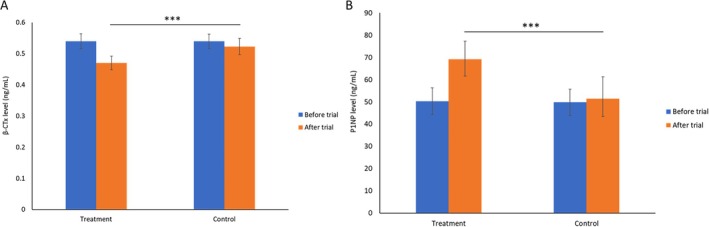
Efficacy of Ruiling capsules on biochemical markers of bone turnover in participants, as assessed by serum β‐CTx (A) and P1NP (B) levels before and after the trial. Participants were allocated into a control group (*n* = 60) and a treatment group (*n* = 60) receiving Ruiling capsules. Data are presented as mean ± SD. Following the trial, the treatment group exhibited significantly lower β‐CTx and higher P1NP levels compared to the control group (***p < 0.01).

The efficacy of Ruiling capsule on improving participants' bone health related biochemical markers was assessed (Figure 5). At baseline, there were no significant differences between groups in β‐CLx (Figure 5A), P1NP (Figure 5B), and 25(OH)D (Extended figure 1) levels (*p* > 0.05). After 32 weeks, the treatment group showed a substantial reduction in β‐CLx (from 539.45 ± 24.83 ng/L to 471.34 ± 22.87 ng/L) and a marked increase in P1NP (from 50.26 ± 6.03 μg/L to 69.18 ± 8.14 μg/L), both changes reaching statistical significance (*p* < 0.01 compared to Control). In contrast, the control group demonstrated only modest changes in these markers (β‐CLx: 540.16 ± 23.46 ng/L to 523.75 ± 25.83 ng/L; P1NP: 49.83 ± 5.97 μg/L to 51.41 ± 9.86 μg/L) with no significant difference. No significant differences were observed in 25‐hydroxyvitamin D levels after treatment between the two groups (Extended figure 1).

#### 3.2.5 Antioxidant Efficacy

**FIGURE 6:**
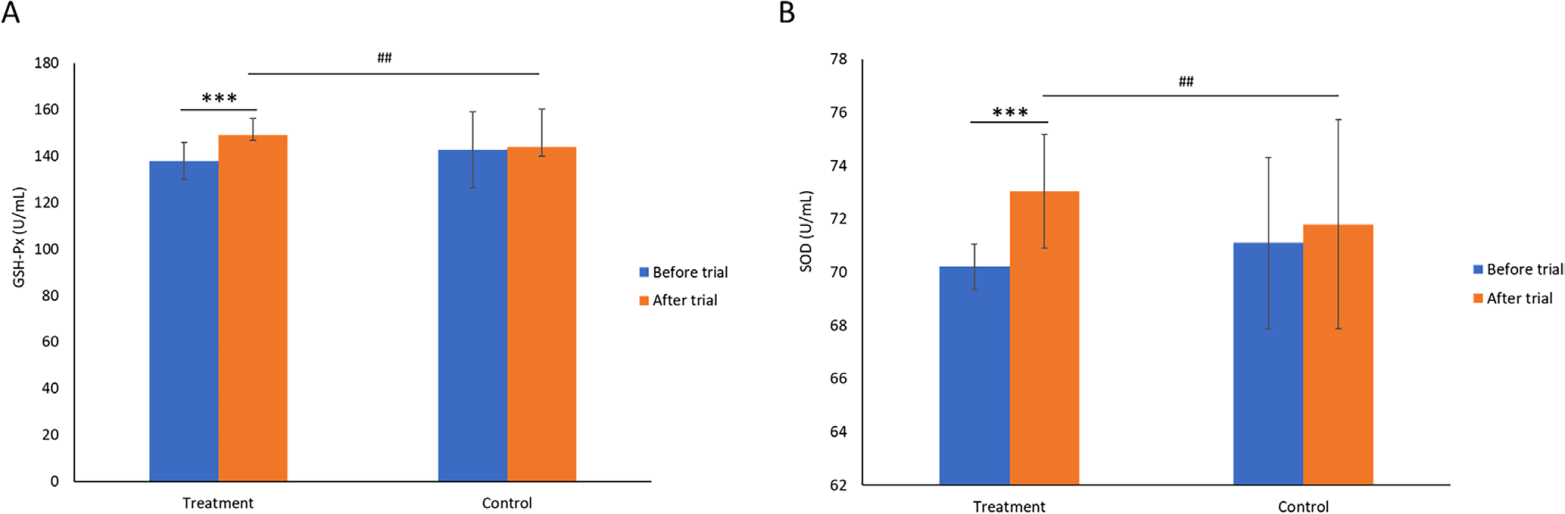
Efficacy of Ruiling capsules on antioxidant enzyme activity, as indicated by serum GSH‐Px (A) and SOD (B) levels in participants before and after the trial. Participants were divided into a treatment group (*n* = 60) receiving Ruiling capsules and a control group (*n* = 60). Data are presented as mean ± SD. Within‐group comparisons for the treatment group before and after the trial are indicated by ***p < 0.01. Between‐group comparisons after the trial are indicated by ##*p* < 0.05.

The antioxidant efficacy of Ruiling capsule is assessed by measuring participants' serum MDA level (Extended figure 2), GSH‐Px (Figure 6A) and SOD activities (Figure 6B). As shown in Extended figure 2, there were no significant differences in MDA concentrations before and after the intervention within either the treatment (7.92 ± 0.21 nmol/mL vs. 7.89 ± 0.28 nmol/mL) or control groups (7.92 ± 0.19 nmol/mL vs. 7.85 ± 0.29 nmol/mL) (*p* > 0.05). Moreover, the comparison of post‐treatment MDA levels between the treatment and control groups did not reveal any statistically significant differences (*p* > 0.05). These findings indicate that the intervention had no appreciable impact on lipid peroxidation as reflected by MDA levels.

Figure 6A demonstrates that, within the treatment group, GSH‐Px activity increased significantly after the intervention (148.91 ± 7.11 U/mL) compared to baseline (137.85 ± 8.08 U/mL) (*p* < 0.01), indicating an enhancement in the endogenous antioxidant system. The control group, however, showed no significant changes in GSH‐Px activity from before to after the intervention (*p* > 0.05).

Between‐group comparisons revealed no significant difference in GSH‐Px activity at baseline (137.85 ± 8.08 U/mL vs. 142.69 ± 16.35 U/mL) (*p* > 0.05). However, after the intervention, the treatment group's GSH‐Px activity (148.91 ± 7.11 U/mL) was significantly higher than that of the control group (143.80 ± 16.30 U/mL) (*p* < 0.05). These results suggest that the tested intervention effectively improves GSH‐Px‐mediated antioxidant capacity.

Figure 6B shows that the trial group experienced a significant increase in SOD activity (U/mL) following the intervention (70.21 ± 0.85 U/mL vs. 73.05 ± 2.14 U/mL) (*p* < 0.01), whereas no significant changes were observed in the control group (71.10 ± 3.22 U/mL vs. 71.81 ± 3.92 U/mL) (*p* > 0.05). When comparing the post‐intervention values between the two groups, the treatment group's SOD activity was markedly higher than that of the control group (73.05 ± 2.14 U/mL vs. 71.81 ± 3.92 U/mL) (*p* < 0.01).

#### 3.2.6 Adverse Events and Safety

Routine blood examinations, liver and kidney function tests revealed no abnormalities in either group before or after the intervention (Table 3).

**TABLE 3:** Summary statistics for blood, urine, and biochemical test.

Parameter	Intervention group (*n* = 60)		Control group (*n* = 60)	
	Before trial	After trial	Before trial	After trial
White blood cells (10^9^/L)	6.04 ± 1.77	5.85 ± 1.04	6.51 ± 1.69	6.30 ± 1.31
Red blood cells (10^12^/L)	4.48 ± 0.61	4.57 ± 0.45	4.48 ± 0.52	4.54 ± 0.42
Platelets (10^9^/L)	215.47 ± 48.04	216.05 ± 38.10	223.31 ± 55.15	228.81 ± 44.29
Hemoglobin (g/L)	135.02 ± 12.87	132.64 ± 11.47	134.28 ± 16.33	132.63 ± 11.35
Total protein (g/L)	75.04 ± 3.55	74.18 ± 3.63	75.38 ± 3.18	75.54 ± 2.76
Albumin (g/L)	48.08 ± 2.22	47.61 ± 2.12	46.62 ± 3.73	45.54 ± 2.55
ALT (U/L)	18.45 ± 7.90	18.40 ± 6.11	18.70 ± 6.56	19.56 ± 6.72
AST (U/L)	19.31 ± 4.29	19.80 ± 3.68	19.67 ± 4.78	19.69 ± 4.53
Urea nitrogen (mmol/L)	5.18 ± 1.15	5.31 ± 1.01	5.25 ± 1.16	5.45 ± 1.00
Creatinine (μmol/L)	61.00 ± 11.10	62.35 ± 9.10	62.89 ± 12.52	61.52 ± 12.45
Total cholesterol (mmol/L)	5.14 ± 0.89	5.18 ± 0.69	5.72 ± 0.27	5.70 ± 0.24
Triglycerides (mmol/L)	1.13 ± 0.38	1.21 ± 0.32	1.83 ± 0.12	1.85 ± 0.13
Glucose (mmol/L)	5.28 ± 0.73	5.07 ± 0.40	5.10 ± 0.49	5.19 ± 0.45
HDL (mmol/L)	1.52 ± 0.36	1.43 ± 0.20	1.31 ± 0.15	1.36 ± 0.19
Uric acid (μmol/L)	284.49 ± 61.00	279.40 ± 46.59	297.52 ± 62.64	296.13 ± 51.91

*Note:* Data are presented as mean ± SD, *n* = 60 in both intervention and control groups.

Self‐reported adverse events (e.g., dry mouth, constipation, mild digestive complaints, fatigue) occurred at a low rate and did not differ significantly between the two groups (*p* > 0.05). There was no statistically significant increase in adverse reactions associated with the treatment regimen in the Observation group compared to the Control group (Table 4).

**TABLE 4:** Summary of reported adverse effect cases during the trial.

	*n*	Dry mouth	Constipation	Indigestion	Fatigue	%
Treatment	60	0	3	2	5	11.1
Control	60	3	4	2	0	10.0

In summary, patients receiving Ruiling capsule in addition to calcium carbonate capsule (treatment group) demonstrated significant improvements in low back pain, BMD, bone turnover, and antioxidant marker profiles without any notable increase in adverse events. This suggests that the treatment may provide a safe and effective option for managing postmenopausal osteoporosis.

## 4 Discussion

This study presents new evidence supporting the efficacy of a combined supplement derived from pilose antler extract and hydroxytyrosol‐rich olive extract (Ruiling capsule) improves bone health in both an animal model of osteoporosis and in postmenopausal women. The results demonstrated beneficial effects on BMD, bone turnover markers, pain reduction, and antioxidant enzyme activity, suggesting a combined effect between pilose antler extract and hydroxytyrosol could offer a promising, non‐pharmacological strategy for the prevention and management of osteoporosis.

Pilose antler has been utilized for centuries in traditional Eastern medicine to support overall health and is considered effective in alleviating clinical symptoms of conditions analogous to osteoporosis (Chen et al. 2015; Liu et al. 2023; Ren et al. 2019; Yun et al. 2020). Previous studies confirmed that dietary supplementation with pilose antler can directly regulate bone growth and remodeling (Hemmings and Song 2004), likely due to its rich and complex composition. Pilose antler contains essential amino acids, collagen, glycosaminoglycans, chondroitin sulphate, proteoglycans, multiple minerals (Ca, P, Fe, Zn), growth factors (e.g., BMPs, FGFs), and various lipids (Sui et al. 2014; Tsujibo et al. 1987). The water‐soluble portion of pilose antler (velvet antler) contains glycosaminoglycans that exhibit growth‐promoting effects on cells (Sunwoo 1998; Sunwoo et al. 1997). Reported by Liu et al., pilose antler polysaccharide and polypeptide extract pose significant inhibitory effects on bone resorption in high‐turnover osteoporosis by modulating MAPK and MMP9 pathways, reducing the expression and activity of ERK1 and JNK (Liu et al. 2023). And peptide extracted from pilose antler was reported to enhance osteoblastogenesis by counteracting TNFα induced suppression of osteoblast differentiation (Liu et al. 2017). In addition, the cartilaginous component of pilose antler contains proteoglycan chondroitin sulphate (which accounts for approximately 90% of total proteoglycans in elk velvet antler) and deproteinated components (Sunwoo et al. 1998). It is generally believed that these cartilage proteoglycans regulate water retention, as well as chondrocyte differentiation and proliferation within cartilage tissue (Alcaide‐Ruggiero et al. 2023). Moreover, four types of collagen (I, II, III, and X) have been identified and immunohistochemically localized in pilose antler (Price et al. 1996). Feng et al. discovered that pilose antler extracts contain a variety of growth factor families, including bone morphogenetic proteins (BMPs) (Feng et al. 1995, 1997) and fibroblast growth factors (FGFs) (Sunwoo et al. 1997). These growth factors possess potent anabolic effects on bone tissue. Specifically, BMPs exert strong local effects on bone formation (Cheng et al. 2001; Fujimura et al. 1995) and the FGF signaling system stimulates osteogenesis, restores trabecular microarchitecture, and enhances fracture repair (Dunstan et al. 1999; Nakamura et al. 1995). Kim et al. further demonstrated that supplementation with pilose antler extract (Kim et al. 2003) in osteoporotic rats maintained well‐connected trabecular structures, effectively preventing bone loss.

Hydroxytyrosol, a phenolic compound found in olives and recognized as safe by EFSA (EFSA Panel on Dietetic Products, Nutrition and Allergies, et al. 2017), also has a long history of human consumption (Fernandez‐Bolanos et al. 2008; Martínez et al. 2018). Hydroxytyrosol has been shown to inhibit osteoclast differentiation through regulation of mitochondrial function and the ERK and JNK signaling pathways (Incani et al. 2010; Zhang et al. 2020), thereby preventing oxidative stress (OS)‐induced damage in pre‐osteoblasts. In addition, hydroxytyrosol mitigates alveolar bone loss, increases bone formation activity, inhibits osteoclast differentiation, and reduces OS levels in mice with periodontitis (Zhang et al. 2021). Both oleuropein (10–100 μM) and hydroxytyrosol (50–100 μM) dose‐dependently inhibit the formation of multinucleated osteoclasts (Hagiwara et al. 2011). Furthermore, these two compounds prevent trabecular bone loss in the femurs of OVX mice (Hagiwara et al. 2011; Puel et al. 2008), while hydroxytyrosol reduces H_2_O_2_ levels in MC3T3‐E1 cells (Gao et al. 2015). These findings suggest that olive polyphenols, including oleuropein and hydroxytyrosol, may play a key role in bone formation and maintenance, and could be used as effective agents to treat osteoporosis‐related symptoms.

Our findings highlight that the combined supplementation (Ruiling capsule) with hydroxytorosol and pilose antler extract provides combinational benefits. The animal studies showed that Ruiling restored BMD and increased bone calcium content beyond levels seen in untreated osteoporotic controls. In the human trial, Ruiling supplementation significantly improved BMD, bone turnover markers, and pain outcomes, indicating that these effects translate into clinically meaningful improvements. The observed antioxidant benefits—included elevated GSH‐Px and SOD activities—suggest that the improvement in bone health may be partially mediated by the mitigation of oxidative stress, reducing the deleterious effects of OS on bone cells and remodeling pathways.

Notably, no significant adverse effects were detected in either the animal or human arms of the study, underscoring the safety and tolerability of Ruiling capsules. This is crucial considering that long‐term pharmacological treatments for osteoporosis may be limited by safety and compliance issues (Cornelissen et al. 2020). By contrast, a functional dietary intervention like Ruiling capsule could serve as a safer, more accessible complement or alternative to conventional drugs, potentially improving patient adherence and quality of life.

Despite these encouraging results, certain limitations persist. Bone health is tightly interwoven with body composition parameters, including fat‐mass index (FMI) and skeletal‐muscle index (SMI) (Villa et al. 2024), which can be incorporated in future trials. Longer‐term clinical studies are needed to confirm the durability of the observed benefits. Mechanistic studies at the molecular and cellular levels would clarify how pilose antler extract and hydroxytyrosol interact to influence specific signaling pathways, inflammatory mediators, and bone cell populations. Larger, more diverse cohorts could validate the generalizability of our findings and refine dosing strategies.

## 5 Conclusion

In conclusion, by leveraging the complex bioactive profile of pilose antler extract—rich in amino acids, glycosaminoglycans, collagen, growth factors—and the potent antioxidative properties of hydroxytyrosol from olive extract, the Ruiling capsule demonstrates a promising intervention for improving bone health in postmenopausal women with osteoporosis. This is the first time that the combinational benefit between pilose antler extract and hydroxytyrosol is investigated in both animal and human subjects. This integrative approach reflects both traditional medical wisdom and modern nutritional and biomedical research.

## Author Contributions

**Xichi Ma:** investigation (equal), methodology (equal), software (equal), validation (equal), visualization (equal), writing – original draft (equal). **Yun Ma:** conceptualization (equal), investigation (equal), methodology (equal), resources (equal), validation (equal), visualization (equal), writing – original draft (equal). **Xilin Ma:** investigation (equal), project administration (equal), software (equal), validation (equal), visualization (equal), writing – original draft (equal). **Zubing Zhang:** data curation (equal), investigation (equal), methodology (equal), project administration (equal), resources (equal), writing – original draft (equal). **Yuan Li:** conceptualization (lead), investigation (equal), supervision (equal), visualization (equal), writing – original draft (equal), writing – review and editing (lead).

## Ethics Statement

This study was conducted according to the guidelines laid down in the Declaration of Helsinki, and all procedures and ethics involving human subjects were approved by the Ethics Committee of Bayi Orthopedic Hospital, Chengdu, China (Document no. 2023(36)). All subjects gave written informed consent before entering the study. All the procedures used in animal tests followed the National Institutes of Health Guidelines for the Care and Use of Animals and were approved by the Department of Pharmacology, West China School of Pharmacy, Sichuan University. This study was registered with the Chinese Food and Drug Administration (CFDA) under identification number GZ04620140034.

## Consent

Informed consent was obtained from all subjects involved in the study.

## Conflicts of Interest

Y.L. served as an unpaid consultant to Hehui Pharmaceutical Technology Co. Ltd in connection with this study. Z.B.Z. is employed by the company Yiping Medical Science & Technology Development Co. Ltd. The remaining authors declare no conflicts of interest.

## Supporting information


**Figure S1.** Efficacy of Ruiling capsule on 25(OH)D level of participants before and after trial.

**Figure S2.** Efficacy of Ruiling capsule on MDA level of participants before and after trial.

## Data Availability

The data that supports the findings of this study are available in the Supporting Information of this article.
