# Supplementary figures and images for: Combination of Pilose Antler Extract and Hydroxytyrosol Enhances Bone Mineral Density in Both Animals and Postmenopausal Women
Source: Food Sci Nutr. 2025 Jun 12;13(6):e70402. doi: 10.1002/fsn3.70402 (PMC12159450; doi:10.1002/fsn3.70402)

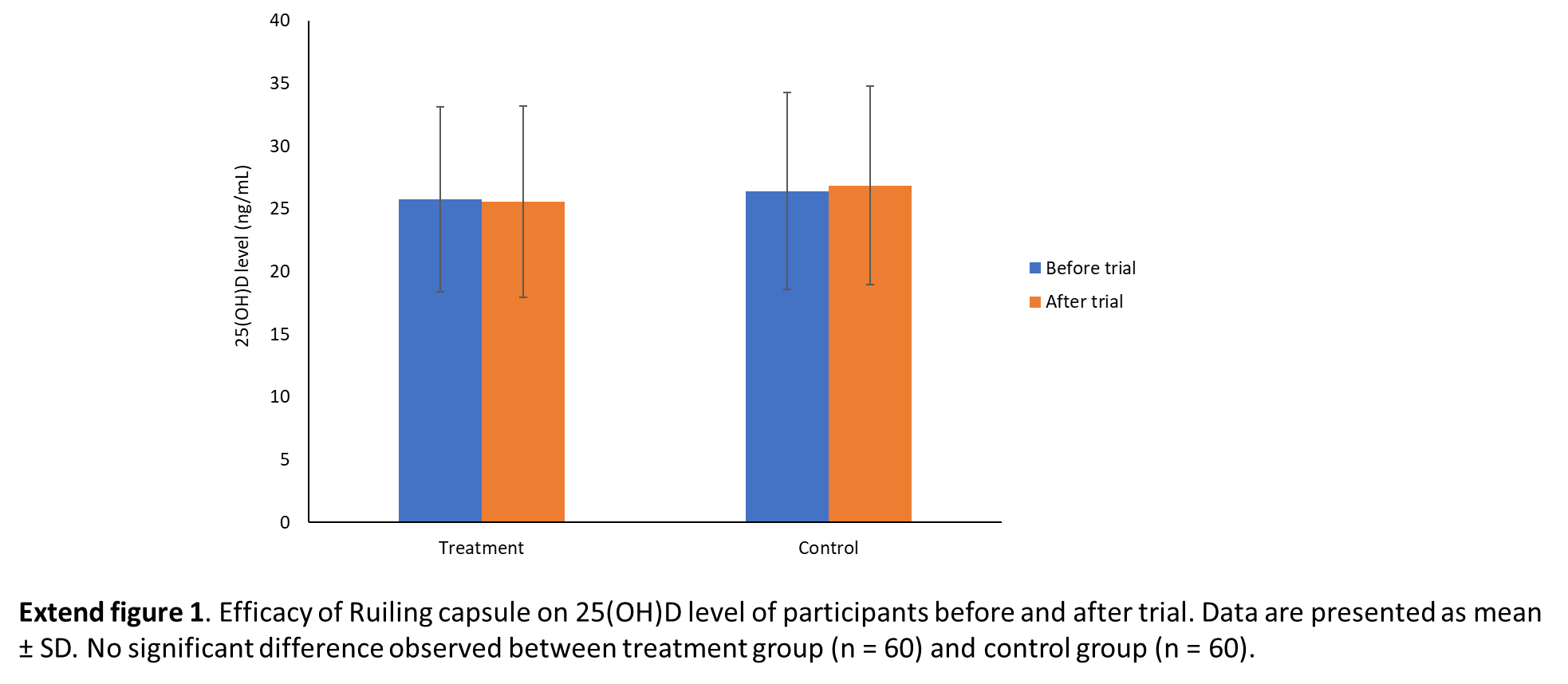

Supplement: Supplementary file 1 — Figure S1. Efficacy of Ruiling capsule on 25(OH)D level of participants before and after trial. [file FSN3-13-e70402-s002.docx]

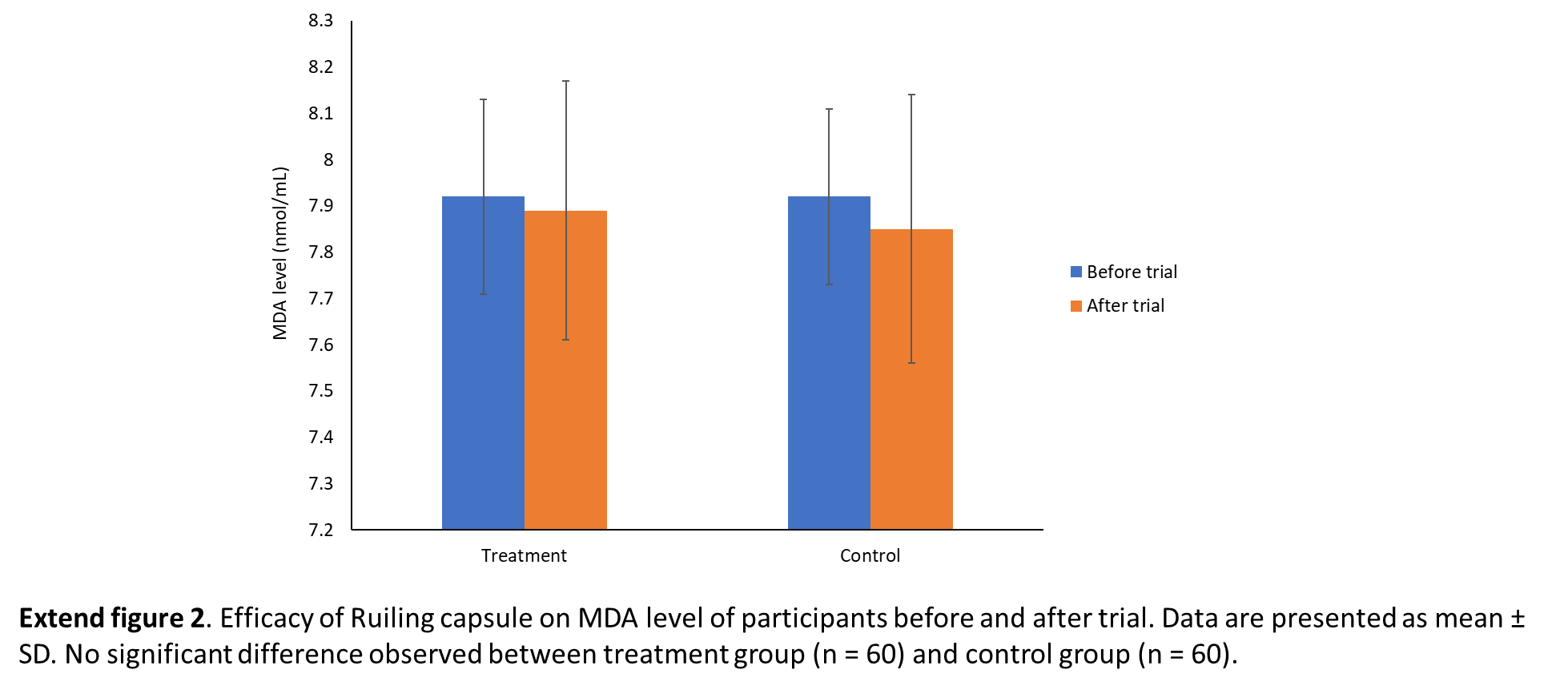

Supplement: Supplementary file 2 — Figure S2. Efficacy of Ruiling capsule on MDA level of participants before and after trial. [file FSN3-13-e70402-s001.docx]
